# Alterations in Metabolic Profile Occur in Normal-Weight and Obese Men during the Ramadan Fast Despite No Changes in Anthropometry

**DOI:** 10.1155/2014/482547

**Published:** 2014-08-06

**Authors:** Jessica McNeil, Mohamed M. Mamlouk, Karine Duval, Alexander Schwartz, Nelson Nardo Junior, Éric Doucet

**Affiliations:** ^1^Behavioural and Metabolic Research Unit (BMRU), School of Human Kinetics, University of Ottawa, Ottawa, ON, Canada K1N 6N5; ^2^Centre de Recherche Clinique Étienne-Le Bel, Centre Hospitalier Universitaire de Sherbrooke, Université de Sherbrooke, Sherbrooke, QC, Canada J1H 5N4; ^3^Department of Nutritional Sciences, Faculty of Medicine, University of Toronto and The Hospital for Sick Children, Toronto, ON, Canada M5S 3E2; ^4^Multiprofessional Nucleus of Obesity Study, Department of Physical Education, State University of Maringa, 87020-900 Maringa, PR, Brazil

## Abstract

We examined the variations in eating behavior, appetite ratings, satiety efficiency, energy expenditure, anthropometric and metabolic profile markers prior to, during as well as 1 and 4 months after Ramadan in normal-weight and obese men. Anthropometric, energy expenditure (indirect calorimetry and accelerometry), metabolic (fasting blood sample), appetite (visual analogue scales), and eating behavior (Three-Factor Eating Questionnaire) measurements were performed in 10 normal-weight (age: 25.2 ± 4.7 years; BMI: 24.4 ± 1.9 kg/m^2^) and 10 obese (age: 27.0 ± 4.5 years; BMI: 34.8 ± 3.7 kg/m^2^) men. The satiety quotient (SQ) was calculated 180 minutes after breakfast consumption. All anthropometric variables, as well as resting and total energy expenditure, were greater in obese compared to normal-weight participants (*P* = 0.02–0.0001). Similarly, obese participants had greater triglycerides, insulin, and homeostatic model assessment-insulin resistance concentrations (*P* = 0.02–0.002). Greater apolipoprotein B, glucose, total cholesterol, and low-density lipoprotein concentrations were noted during Ramadan (*P* = 0.04–0.0001). Dietary restraint scores were also greater during Ramadan (*P* = 0.0001). No differences in anthropometry, other metabolic profile markers, energy expenditure, appetite ratings, and SQ were noted across sessions. Lastly, changes in anthropometric measurements correlated with delta metabolic profile markers, as well as changes in disinhibition eating behavior trait and dietary restraint scores. The Ramadan fast led to increases in certain metabolic profile markers despite no changes in appetite and anthropometry.

## 1. Introduction

Many studies have assessed the effects of the Ramadan fast on different metabolic and anthropometric parameters, but have noted conflicting results. More specifically, some studies noted decreases in body weight and body mass index (BMI) during the Ramadan fast [1–5], while others noted no significant differences in these same variables when assessed prior to, during, and/or following the Ramadan fast [6–10]. Similarly, certain studies noted no significant differences in fat mass and fat-free mass during the fast [9, 10], whereas another study observed a significant decrease in fat mass and no change in fat-free mass during the Ramadan fast [3].

With regards to metabolic profile markers, some studies noted improvements in metabolic profiles (i.e., lower levels of fasting cholesterol, glucose, and triglyceride) during the Ramadan fast [5, 11–14]. Conversely, other studies noted either no significant difference in these same parameters across time [2, 6, 8] or significant increases in total cholesterol [4] during the Ramadan fast. To our knowledge, only 1 study has previously assessed potential variations in resting energy expenditure (REE) during the Ramadan fast and noted no changes in this variable [10]. Significant time of day, but not between session (i.e., prior to, during, and after Ramadan fast) variations in physical activity energy expenditure (PAEE) were noted [9, 10]. Similarly, only 1 study has previously assessed variations in appetite ratings prior to, during, and 21 days after Ramadan fast and observed significant differences throughout the day during the fast, as well as decreases in hunger ratings as the Ramadan fast progressed in women, whereas hunger ratings in men remained the same throughout the Ramadan fast [7].

While many studies have assessed potential variations in different metabolic and anthropometric parameters prior to, during, and/or up to 1 month after Ramadan, none have reassessed these variables four months later in order to evaluate whether potential changes in these variables may persist over a longer period of time. Furthermore, the assessment of different appetite, anthropometric and metabolic profile markers in normal-weight and obese individuals across the Ramadan fast is scarce, with only 1 study previously comparing the changes in body weight and the metabolic profile of normal-weight and obese men during the Ramadan fast [15]. Hence, the objective of the present study was to evaluate the potential variations in eating behavior, appetite ratings, the satiety efficiency (via the satiety quotient), total energy expenditure (TEE), anthropometry, and different metabolic profile markers prior to, during as well as 1 and 4 months after Ramadan. Furthermore, this study assessed whether variations in these variables differed between normal-weight (BMI ≤ 27 kg/m²) and obese (BMI ≥ 30 kg/m²) men. We hypothesized that decreases in body weight, waist circumference, and fat mass would be observed during the Ramadan fast, which would coincide with a decrease in TEE and increases in feelings of dietary restraint and desire to eat/hunger ratings during this fast. We further hypothesized that improvements in markers of metabolic profile would be observed during the Ramadan fast. Lastly, we hypothesized that these effects would be greater in obese compared to normal-weight individuals.

## 2. Materials and Procedures 

### 2.1. Participants

Ten normal-weight (BMI ≤ 27 kg/m²) and ten obese (BMI ≥ 30 kg/m²) men took part in the present study. These BMI cut-off points were based on those established by The National Institutes of Health Consensus Development Panel on the Health Implications of Obesity [16], which classify “overweight adult males” as having a BMI ≥ 27.8 kg/m². Participants were Muslim men between the ages of 20–35 years who fast during the month of Ramadan. They were also nonsmokers and weight stable (±4 kg) for at least 6 months prior to the start of data collection and did not have heart problems or diabetes. Data collection for this study took place in 2008, at which time the Ramadan fast started on Monday, September 1st. This study was conducted according to the guidelines laid down in the Declaration of Helsinki and the University of Ottawa and Montfort Hospital ethics committees approved all procedures involving human participants. Written informed consent was obtained from all participants.

### 2.2. Design and Procedure

Different measurements were taken at baseline (before the onset of Ramadan; session 1), 2 weeks into the Ramadan fast (session 2), 1 week before the end of the Ramadan fast (session 3), 3-4 weeks following the end of the Ramadan fast (session 4), and finally 16–20 weeks following the end of the Ramadan fast (session 5). Measurements were taken over 5 hours during sessions 1, 4, and 5. These sessions began at 7:30 a.m. for all participants. As for sessions 2 and 3, which lasted 1.5 hours each, measurements were taken from a maximum of 5 participants over the course of 1 morning, and their start times were staggered between 8:30 a.m. and 10:30 a.m. (i.e., 2, 2, and 1 participants started measurements at 8:30 a.m., 9:30 a.m. and 10:30 a.m., respectively, during sessions 2 and 3). Anthropometric measurements were performed during each session. REE and the thermic effect of food (TEF) were assessed during sessions 1, 4, and 5. Habitual PAEE was assessed for seven days during sessions 1, 2, 4, and 5. The participants' appetite sensations were recorded every 30 minutes for 3 hours following the consumption of a standardized breakfast with visual analogue scales (VAS) [17] during sessions 1 and 5. The Three-Factor Eating Questionnaire (TFEQ) [17] was administered during sessions 1, 3, and 5 to assess certain cognitive determinants of eating behavior. Lastly, a single blood sample was taken during sessions 1, 2, and 5 to assess total cholesterol, high-density lipoprotein cholesterol (HDL-C), low-density lipoprotein cholesterol (LDL-C), triglyceride, glucose, insulin, and apolipoprotein B (Apo-B) levels when participants were fasting (i.e., 12-hour overnight fast prior to measurements during sessions 1 and 5; 8-hour fast prior to measurements during session 2). A fasting blood sample was taken during session 4; however this was only completed in 13 participants. Therefore, we decided to remove these data from analyses.

### 2.3. Anthropometric Measurements

Participants were weighed to the nearest 0.1 kg with a BWB-800AS digital scale. Standing height was measured to the nearest centimeter using a wall stadiometer, Tanita HR-100 height rod, without shoes (Tanita Corporation of America, Inc.). Body composition (total fat mass and total fat-free mass) was assessed with DXA (Lunar Prodigy, General Electric, Madison, WI, USA). The coefficient of variation and correlation for body fat percentage assessed in 12 healthy participants tested in our laboratory were 1.8% and *r* = 0.99, respectively.

### 2.4. Energy Expenditure Measurements

REE was evaluated with indirect calorimetry (Deltatrac II metabolic cart, Sensor Medics Corporation, Yorba Linda, California, USA) following an overnight fast. The TEF was evaluated with indirect calorimetry for 1.5 hours over a 3-hour time span (i.e., 3 rotations of 30 minutes of measurements with 30 minutes of no measurements) following the consumption of a standardized breakfast. The coefficient of variation and correlation for REE measured with the Deltatrac II metabolic cart were 2.3% and *r* = 0.98, respectively. PAEE was predicted with an accelerometer (Actical Accelerometer, Bio-Lynx Scientific Equipment, Montreal, Quebec, Canada) for 7 days under free-living conditions. During sessions 1, 4, and 5, the values for REE, TEF, and PAEE were added to obtain TEE for each of these sessions.

### 2.5. Cognitive Determinants of Eating Behavior

The TFEQ [18] was used to evaluate certain cognitive determinants of eating behavior. More specifically, this 51-item questionnaire measures the degree of cognitive dietary restraint, disinhibition eating behavior trait, and feelings of hunger.

### 2.6. Standardized Breakfast, Appetite Measurements, and Calculation of the Satiety Quotient

A standardized breakfast was served at 9:30 a.m. during sessions 1, 4, and 5. This breakfast contained whole-wheat bread (80 grams), peanut butter (36 grams), strawberry jam (32 grams), cheddar cheese (21 grams) and orange juice (250 grams), and had a caloric content of 592 kilocalories and a food quotient of 0.89 (57% carbohydrates, 13% proteins and 30% lipids). The participants were instructed to consume the entire meal in 15 minutes. Appetite sensations were recorded with VAS before, immediately after (time 0), and every 30 minutes (30, 60, 90, 120, 150, and 180 minutes) for 3 hours following breakfast consumption. The 150 mm VAS [16] was used to answer 4 specific questions that quantify subjective appetite sensations: desire to eat (“How strong is your desire to eat?”), hunger (“How hungry do you feel?”), fullness (“How full do you feel?”), and prospective food consumption (PFC) (“How much food do you think you could eat?”). The measurements before breakfast were considered as fasting measurements, whereas postmeal areas under the curve (AUC) were calculated with the trapezoid method [19]. Appetite ratings at 0, 30, 60, 90, 120, 150, and 180 minutes were included in the calculation of postmeal AUC.

The satiety quotient (SQ), which is a valid marker of satiety efficiency in response to a standardized meal [20], was calculated for each appetite sensation at 180 minutes following breakfast consumption with the following equation adapted from Green et al. [21]:

(1) SQ(mm/100 kcal) =[fasting  appetite  sensation(mm)−mean  post  meal  appetite  sensation(mm)]  ×(energy  content  of  the  test  meal(kcal))−1  ×100.

It is important to note that SQ calculation for the fullness rating is reversed (i.e., mean postmeal fullness rating − fasting fullness rating). The mean SQ, determined as the mean value of SQ scores for the 4 appetite sensations, was also calculated. The SQ calculation has a good reliability when assessed under controlled laboratory conditions, with the intraclass correlation coefficient value (*r* = 0.67) being greatest for mean SQ [22]. The possible range for SQ values in the current study is between −25.3 and 25.3 mm/100 kcal. A higher SQ indicates a greater satiety response to the standardized meal [23].

### 2.7. Blood Samples

A single blood sample was drawn from the antecubital vein of the nondominant arm following an overnight fast to determine plasma levels of total cholesterol, HDL-C, LDL-C, triglyceride, glucose, insulin, and Apo-B. Blood samples were placed into a tube containing Ethylenediaminetetraacetic acid (EDTA) and were centrifuged at 3500 rpm at 4°C immediately after the blood was drawn and stored at −80°C until assayed. Plasma insulin concentrations were determined by radioimmunoassay using ^125^I-labeled human insulin and a human insulin antiserum (Millipore, St. Charles, Missouri). Plasma glucose concentrations were determined with spectrophotometric analysis after converting the glucose to glucose-6-phosphate via hexokinase (Sigma-Aldrich Canada Ltd., Oakville, Ontario, Canada; Fisher Scientific Limited, Nepean, Ontario, Canada). Homeostasis model assessment-insulin resistance (HOMA-IR) was estimated with the following equation: HOMA-IR = (fasting glucose ∗ fasting insulin)/22.5. Total cholesterol, HDL-C, and triglyceride levels were analyzed with the Vitros 950 immunoassay analyzer (Ortho Clinical Diagnostics; Johnson & Johnson Company, Markham, Ontario, Canada) at a wavelength of 540 nanometers. These same variables were used in the Friedewald formula to determine LDL-C levels [24]. Apo-B levels were assessed by immunonephelometry on an Image Analyzer (Beckman-Coulter, Villepinte, France).

### 2.8. Statistical Analyses

Statistical analyses were performed using SPSS (version 17.0; SPSS Inc., Chicago, IL). Two-way repeated measures ANOVA with BMI status (normal-weight and obese) as the between-subject factor were used to determine the main effects of time (baseline, during the Ramadan fast, and after Ramadan fast) on anthropometric parameters (body weight, waist circumference, body fat mass and body fat-free mass), TEE, REE, TEF, PAEE, and metabolic profile markers (total cholesterol, HDL-C, LDL-C, triglyceride, glucose, insulin, Apo-B and HOMA-IR), as well as TFEQ scores (dietary restraint, disinhibition eating behavior trait and feelings of hunger). Paired* t*-tests were used to assess the main effects of time (sessions 1 and 5) on fasting appetite ratings and postmeal AUC appetite ratings, as well as desire to eat, hunger, fullness, PFC, and mean SQ. The Wilcoxon Signed Ranks Test was used to assess potential differences across time for variables that were not normally distributed according to the Shapiro-Wilk test. Post hoc tests with Bonferroni adjustments were used to assess potential significant differences.

Bivariate Spearman correlations were used to assess whether the changes in metabolic profile markers (delta sessions 1-2, 1–5, and 2–5) and TFEQ scores (delta sessions 1–3, 1–5, and 3–5), as well as fasting and postmeal AUC appetite ratings and SQ (delta sessions 1–5), were associated with changes in anthropometric parameters between these same sessions. Data are presented as means ± standard deviations, and effects with *P* values <0.05 were considered statistically significant.

## 3. Results

The variations in anthropometric parameters across sessions and between BMI groups are presented in Table 1. No significant differences in body weight, waist circumference, total fat-free mass, and total fat mass were noted across time. As expected, all of these variables were significantly greater in obese compared to normal-weight participants. No significant differences in REE, TEF, PAEE and TEE were noted across sessions, whereas only REE and total EE were significantly greater in obese compared to normal-weight participants (Table 1). Similarly, no differences in disinhibition eating behavior trait and susceptibility to hunger were noted across time; however, dietary restraint scores were significantly greater during session 3 (during Ramadan fast) compared to sessions 1 (*P* = 0.002) and 5 (*P* = 0.0001) (baseline and after Ramadan fast) (Table 1). No significant time ∗ group interactions were noted for all of these variables (Table 1).

No differences in HOMA-IR, insulin, HDL-C, and triglyceride levels were noted across sessions (Table 2). However, glucose, Apo-B, total cholesterol, and LDL-C levels were significantly greater during the Ramadan fast (session 2) compared to baseline (session 1; *P* = 0.001 for LDL-C, *P* = 0.01 for total cholesterol, *P* = 0.01 for Apo-B) and/or after Ramadan fast (session 5; *P* = 0.001 for LDL-C, *P* = 0.03 for total cholesterol, *P* = 0.01 for glucose). As expected, triglyceride, insulin, and HOMA-IR were greater in obese compared to normal-weight participants (Table 2). No significant time ∗ group interactions were noted for all of these variables (Table 2). No significant differences in fasting and postmeal AUC were noted for all appetite ratings (results not shown). Similarly, no significant differences in SQ for all appetite ratings were noted across sessions in both groups (results not shown).

Table 3 summarizes the statistically significant correlations between changes in anthropometric parameters and changes in metabolic profile markers with delta TFEQ scores, delta fasting and postmeal AUC appetite ratings, as well as delta SQ between different sessions. Changes in body weight, waist circumference, and total fat mass were positively associated with changes in different metabolic profile markers. Conversely, changes in total fat-free mass were negatively associated with changes in total cholesterol and HOMA-IR. Lastly, changes in disinhibition eating behavior trait were positively associated with delta fat mass between sessions 1 and 3, whereas changes in dietary restraint scores were negatively associated with delta fat-free mass between sessions 1 and 5. No other significant correlations were noted between the changes in anthropometric parameters with the changes in metabolic profile markers, TFEQ scores, SQ, and appetite ratings (results not shown).

## 4. Discussion 

To our knowledge, this is the first study to evaluate eating behavior, appetite ratings, satiety efficiency, TEE, anthropometry, and different metabolic profile markers prior to, during as well as 1 and 4 months following the end of the Ramadan fast in normal-weight and obese men. Collectively, our results demonstrated that no changes in anthropometry, energy expenditure, appetite ratings, and SQ occurred across time, which does not support our initial hypothesis. Significant increases in Apo-B, glucose, total cholesterol, and LDL-C levels were noted during the Ramadan fast, which also does not support our hypotheses. Greater dietary restraint scores were noted at this time. Changes in body weight, waist circumference, and fat mass at baseline, during Ramadan, and 16–20 weeks following the Ramadan fast were positively associated with changes in most metabolic profile markers, whereas changes in fat-free mass were negatively associated with changes in total cholesterol and HOMA-IR. Lastly, delta disinhibition eating behavior trait scores were positively associated with changes in fat mass between baseline and the Ramadan fast, whereas delta dietary restraint scores were negatively associated with changes in fat-free mass between baseline and 16–20 weeks after Ramadan.

The noted increases in Apo-B, glucose, LDL-C, and total cholesterol levels are supported by 1 other study [4], which assessed metabolic profile markers at the start and at the end of the Ramadan fast in 24 normal-weight men and women, and noted significantly greater total cholesterol levels at the end* versus* the start of the fast. Additionally, the current study observed positive correlations between the changes in body weight, waist circumference, and fat mass with changes in most metabolic profile markers between the Ramadan fast and baseline/16–20 weeks after Ramadan fast, as well as between baseline and 16–20 weeks after Ramadan fast. The changes in total cholesterol levels and HOMA-IR were also negatively correlated with changes in fat-free mass between the Ramadan fast and baseline/16–20 weeks after Ramadan fast. These novel findings suggest that individuals with greater increases in body weight, waist circumference, and fat mass, as well as decreases in fat-free mass, may experience greater negative alterations to their metabolic profile. Ziaee et al. [2] also measured correlations between the changes in body weight with changes in many metabolic profile markers at baseline and on the 26th day of the Ramadan fast in 81 medical students and only observed a significant positive association between delta glucose levels and body weight change.

Ünalacak et al. [15] previously evaluated the changes in many anthropometric and metabolic profile markers prior to and following the month of Ramadan fast in normal-weight and obese Turkish men and observed a significant decrease in body weight, BMI, HOMA-IR, and fasting blood glucose in the obese participants only. Conversely, the present study noted no significant differences in anthropometry and the metabolic profile of normal-weight and obese participants across time, in addition to noting either adverse or no changes in anthropometry and metabolic profile markers across time. This discrepancy in results may be in part explained by the time of assessment (i.e., assessments at baseline, during, and after Ramadan fast in the present study* versus* assessments before and following the Ramadan fast in [15]). Furthermore, yearly differences associated with the practice of Ramadan may in part explain the differences in results noted between studies, as the month of Ramadan can be held during different seasons across different years, which leads to a daily fasting time that varies between 10 and 18 hours depending on the amount of sunlight during each season [25]. Regional and cultural differences (e.g., religious activities and variations in daily work schedules) may also have a substantial impact on the different activities performed during the Ramadan fast in different countries and should thus be taken into consideration when comparing the results discussed by different studies [25].

No differences in REE, TEF, PAEE and TEE across time in normal-weight and obese participants were noted in the present study. These results are in agreement with other studies that also measured REE and PAEE in normal-weight men and women [1, 9, 10]. Leibel et al. [26] previously observed that variations in body weight within a ±10% range are associated with little change in energy expenditure variables. These results thus suggest that the absence of differences in energy expenditure measurements during the Ramadan fast in the present study is in line with the lack of change in body weight and body composition observed during this same period.

As for eating behavior, dietary restraint scores were significantly greater during the Ramadan fast, which may be expected as increases in dietary restraint scores were also found to be greater in regular dieters [18]. Furthermore, greater increases in disinhibition eating behavior trait between baseline and during the Ramadan fast were associated with greater increases in fat mass. These results thus suggest that individuals with greater increases in disinhibition eating behavior trait during the Ramadan fast may be at an increased risk for fat mass gains over time or* vice versa* as we cannot infer causality from these results. Greater increases in dietary restraint scores between baseline and 16–20 weeks after Ramadan were also associated with greater decreases in fat-free mass. These results are supported by a study [27] in which lower dietary restraint scores were associated with greater fat-free mass in freshmen University students. Hence, it may be suggested that individuals with a greater cognitive dietary restraint throughout the Ramadan fast may experience a greater loss in fat-free mass. However, these results must be interpreted with caution, as this significant correlation does not allow us to draw a cause-and-effect relationship between the changes in dietary restraint scores and those in fat-free mass.

Lastly, no significant differences in appetite ratings and all measured SQ variables were noted across time in normal-weight and obese participants in the present study. To our knowledge, only one other study has previously assessed variations in appetite ratings throughout the day during the Ramadan fast, during the Ramadan feast (day 31; the day after the end of the Ramadan fast), and within 1 month after Ramadan [7]. This study noted significant increases in daily hunger ratings during the Ramadan fast and a significant sex ∗ time interaction between sessions (i.e., women saw a decrease in hunger ratings as the Ramadan fast progressed, whereas men maintained similar hunger levels throughout the fast). Taken together, the results from the present study add to those previously noted by Finch et al. [7], supporting the idea that no differences in appetite ratings seem to occur in response to the Ramadan fast in men, despite the significant variations in daily feelings of hunger that were previously noted by Finch et al. [7].

The present findings are limited to a small sample size of normal-weight and obese men living in Canada, which limits generalizability to other populations (e.g., women and residents of other countries). Not all measurements were taken during each session, and the start of measurements during sessions 2 and 3 did vary between participants (range between 8:30 a.m. and 10:30 a.m.). Measurements of energy intake were taken with 7-day food diaries at baseline, during Ramadan and 16–20 weeks after Ramadan; however, based on the high degree of nonresponders (e.g., 7 participants did not complete the food diaries after Ramadan) and underreporters (e.g., underreporting was noted in 3 participants of the remaining 13 during session 5) in this cohort, we decided not to include these data in the analyses. Likewise, it is possible that potential differences in the types of meals consumed prior to, during, and following the Ramadan fast may have an impact on blood measurements, even though these measurements were taken in participants when fasted (i.e., following a 12-hour overnight fast in sessions 1 and 5, and an 8-hour fast in session 2). Only 1-day assessments of the measured outcomes were performed during each session, which does not account for normal day-to-day variations in these variables. Lastly, the correlations computed between changes in appetite ratings, TFEQ scores, anthropometry, and metabolic profile markers cannot infer causality.

In conclusion, we observed significant increases in Apo-B, glucose, total cholesterol, and LDL-C concentrations during the Ramadan fast in normal-weight and obese men. Dietary restraint scores were also greater during Ramadan. Lastly, changes in anthropometric parameters were related to changes in metabolic profiles, dietary restraint, and disinhibition eating behavior trait scores. Future studies would be needed to assess the longer-term effects of the Ramadan fast on measures of energy expenditure, anthropometry, appetite, and metabolic profiles, as changes in some of these parameters may only occur over many years.

## Figures and Tables

**Table 1:** Variations in anthropometry, energy expenditure, and Three-Factor Eating Questionnaire variables across time, and between BMI status groups.

	Session 1		Session 2		Session 3		Session 4		Session 5		Repeated-measures ANOVA		
	Mean	SD	Mean	SD	Mean	SD	Mean	SD	Mean	SD	Time effect	Group effect	Time ∗ group interaction
Body weight (kg)													
Normal-weight	72.8	9.2	72.2	9.4	71.8	9.4	72.1	9.6	72.9	10.5	*P* = 0.16	*P* = 0.0001	*P* = 0.39
Obese	107.9	15.7	106.0	15.1	105.3	15.4	105.0	15.1	105.7	14.8			
Waist circumference (cm)													
Normal-weight	82.6	6.3	82.3	7.2	83.3	7.8	82.6	7.6	84.8	8.4	*P* = 0.29	*P* = 0.0001	*P* = 0.15
Obese	113.3	10.8	110.7	11.1	111.9	11.2	110.4	12.2	110.8	11.6			
Fat mass (kg)													
Normal-weight	17.6	4.2	17.7	4.6	17.6	4.5	17.4	4.9	18.1	5.4	*P* = 0.39	*P* = 0.0001	*P* = 0.27
Obese	39.1	10.3	39.2	10.4	37.7	9.6	37.8	9.7	36.9	9.1			
Fat-free mass (kg)													
Normal-weight	55.1	5.9	54.3	5.7	54.1	5.8	54.6	5.7	54.0	6.5	*P* = 0.06	*P* = 0.001	*P* = 0.08
Obese	68.2	9.4	66.3	9.2	67.1	9.5	67.0	8.6	68.6	10.0			
REE (kcal)													
Normal-weight	1605	197	NA	NA	NA	NA	1559	244	1592	260	*P* = 0.24	*P* = 0.001	*P* = 0.82
Obese	2132	389	NA	NA	NA	NA	2076	336	2143	317			
TEF (kcal)													
Normal-weight	41	13	NA	NA	NA	NA	44	17	44	16	*P* = 0.60	*P* = 0.88	*P* = 0.84
Obese	43	16	NA	NA	NA	NA	44	17	45	19			
PAEE (kcal)													
Normal-weight	796	195	796	186	NA	NA	777	182	794	227	*P* = 0.97	*P* = 0.14	*P* = 0.86
Obese	1015	444	945	310	NA	NA	1015	362	984	367			
Total EE (kcal)													
Normal-weight	2442	320	NA	NA	NA	NA	2380	363	2431	443	*P* = 0.60	*P* = 0.002	*P* = 0.99
Obese	3190	714	NA	NA	NA	NA	3135	511	3172	513			
Dietary restraint score													
Normal-weight	9	3	NA	NA	12	3	NA	NA	9	2	*P* = 0.0001	*P* = 0.29	*P* = 0.29
Obese	8	4	NA	NA	9	4	NA	NA	8	4			
Disinhibition eating behavior trait score													
Normal-weight	6	2	NA	NA	5	2	NA	NA	6	2	*P* = 0.25	*P* = 0.27	*P* = 0.60
Obese	6	2	NA	NA	7	3	NA	NA	6	3			
Feelings of hunger score													
Normal-weight	7	3	NA	NA	7	2	NA	NA	6	2	*P* = 0.30	*P* = 0.91	*P* = 0.32
Obese	6	2	NA	NA	7	3	NA	NA	6	3			

Session 1: baseline/before the onset of Ramadan.

Session 2: 2 weeks into the Ramadan fast.

Session 3: 1 week before the end of the Ramadan fast.

Session 4: 3-4 weeks following the end of the Ramadan fast.

Session 5: 16–20 weeks following the end of the Ramadan fast.

Note: SD: standard deviation; kg: kilogram; cm: centimeter; REE: resting energy expenditure; kcal: kilocalorie; TEF: thermic effect of food; PAEE: physical activity energy expenditure; EE: energy expenditure.

NA signifies that this measurement was not taken during this session.

**Table 2:** Variations in metabolic parameters across time and between BMI status groups.

	Session 1		Session 2		Session 5		Repeated-measures ANOVA		
	Mean	SD	Mean	SD	Mean	SD	Time effect	Group effect	Time ∗ group interaction
Total cholesterol (mmol/L)									
Normal-weight	4.31	0.68	4.38	0.78	4.29	0.65	*P* = 0.03	*P* = 0.06	*P* = 0.12
Obese	4.75	0.75	5.20	0.56	4.57	0.55			
HDL-C (mmol/L)									
Normal-weight	1.19	0.21	1.17	0.21	1.22	0.27	*P* = 0.50	*P* = 0.11	*P* = 0.38
Obese	1.01	0.17	1.10	0.17	1.07	0.16			
LDL-C (mmol/L)									
Normal-weight	2.69	0.61	2.87	0.74	2.60	0.78	*P* = 0.0001	*P* = 0.18	*P* = 0.06
Obese	2.99	0.65	3.51	0.43	2.70	0.40			
Triglyceride (mmol/L)									
Normal-weight	0.96	0.46	0.77	0.16	1.02	0.62	*P* = 0.05	*P* = 0.01	*P* = 0.88
Obese	1.65	1.10	1.35	0.83	1.75	1.17			
Glucose (mmol/L)									
Normal-weight	4.6	0.3	4.8	0.3	4.5	0.4	*P* = 0.01	*P* = 0.05	*P* = 0.83
Obese	5.0	0.7	5.2	0.5	4.8	0.6			
Insulin (pmol/L)									
Normal-weight	26.7	9.2	25.8	8.9	28.8	12.2	*P* = 0.57	*P* = 0.002	*P* = 0.46
Obese	73.1	60.6	56.4	32.5	51.0	19.7			
Apo-B (g/L)									
Normal-weight	0.77	0.13	0.80	0.16	0.80	0.17	*P* = 0.04	*P* = 0.05	*P* = 0.16
Obese∗	0.90	0.19	1.00	0.16	0.90	0.17			
HOMA-IR (units)									
Normal-weight	5.4	1.8	5.5	2.0	5.9	2.7	*P* = 0.25	*P* = 0.02	*P* = 0.19
Obese	17.7	18.5	13.5	8.4	10.9	4.7			

Session 1: baseline/before the onset of Ramadan.

Session 2: 2 weeks into the Ramadan fast.

Session 3: 1 week before the end of the Ramadan fast.

Session 4: 3-4 weeks following the end of the Ramadan fast.

Session 5: 16–20 weeks following the end of the Ramadan fast.

*n = 9 obese participants.

Note: SD: standard deviation; mmol: millimole; L: litre; HDL-C: high-density lipoprotein cholesterol; LDL-C: low-density lipoprotein cholesterol; pmol: picomole; Apo-B: apolipoprotein B; g: gram; HOMA-IR: homeostatic model assessment-insulin resistance.

**Table 3:** Statistically significant correlations between changes in anthropometric parameters with delta metabolic markers, delta disinhibition eating behavior trait score, and delta dietary restraint score between different sessions.

	Body weight (kg)	Waist circumference (cm)	Fat mass (kg)	Fat-free mass (kg)
Total cholesterol (mmol/L)				
Delta sessions 1 and 2				*r* = −0.55; *P* = 0.01
Delta sessions 1 and 5		*r* = 0.49; *P* = 0.03		
LDL (mmol/L)				
Delta sessions 1 and 5		*r* = 0.48; *P* = 0.03		
Triglyceride (mmol/L)				
Delta sessions 1 and 5		*r* = 0.49; *P* = 0.03		
Glucose (mmol/L)				
Delta sessions 1 and 5	*r* = 0.62; *P* = 0.004	*r* = 0.46; *P* = 0.04	*r* = 0.54; *P* = 0.01	
Insulin (pmol/L)				
Delta sessions 1 and 5	*r* = 0.54; *P* = 0.01		*r* = 0.50; *P* = 0.02	
Delta sessions 2 and 5		*r* = 0.49; *P* = 0.03	*r* = 0.47; *P* = 0.04	
APB (g/L)				
Delta sessions 2 and 5		*r* = 0.52; *P* = 0.02		
HOMA-IR (units)				
Delta sessions 1 and 5	*r* = 0.54; *P* = 0.02	*r* = 0.47; *P* = 0.04	*r* = 0.50; *P* = 0.03	
Delta sessions 2 and 5				*r* = −0.55; *P* = 0.01
Disinhibition eating behavior trait score				
Delta sessions 1 and 3			*r* = 0.46; *P* = 0.04	
Dietary restraint score				
Delta sessions 1 and 5				*r* = −0.49; *P* = 0.03

Note: kg: kilogram; cm: centimeter; mmol: millimole; L: litre; pmol: picomole; g: gram; HOMA-IR: homeostatic model assessment-insulin resistance; mm: millimeter.

## References

[1] Al-Hourani HM, Atoum MF (2007). Body composition, nutrient intake and physical activity patterns in young women during Ramadan. *Singapore Medical Journal*.

[2] Ziaee V, Razaei M, Ahmadinejad Z (2006). The changes of metabolic profile and weight during Ramadan fasting. *Singapore Medical Journal*.

[3] Sweileh N, Schnitzler A, Hunter GR, Davis B (1992). Body composition and energy metabolism in resting and exercising muslims during Ramadan fast. *Journal of Sports Medicine and Physical Fitness*.

[4] Fedail SS, Murphy D, Salih SY, Bolton CH, Harvey RF (1982). Changes in certain blood constituents during Ramadan. *The American Journal of Clinical Nutrition*.

[5] Adlouni A, Ghalim N, Benslimane A, Lecerf JM, Saïle R (1997). Fasting during Ramadan induces a marked increase in high-density lipoprotein cholesterol and decrease in low-density lipoprotein cholesterol. *Annals of Nutrition and Metabolism*.

[6] Akanji AO, Mojiminiyi OA, Abdella N (2000). Beneficial changes in serum apo A-1 and its ratio to apo B and HDL in stable hyperlipidaemic subjects after Ramadan fasting in Kuwait. *European Journal of Clinical Nutrition*.

[7] Finch GM, Day JEL, Welch DA, Rogers PJ (1998). Appetite changes under free-living conditions during Ramadan fasting. *Appetite*.

[8] Maislos M, Khamaysi N, Assali A, Abou-Rabiah Y, Zvili I, Shany S (1993). Marked increase in plasma high-density-lipoprotein cholesterol after prolonged fasting during Ramadan. *The American Journal of Clinical Nutrition*.

[9] Racinais S, Périard JD, Li CK, Grantham J (2012). Activity patterns, body composition and muscle function during Ramadan in a Middle-East Muslim Country. *International Journal of Sports Medicine*.

[10] El Ati J, Beji C, Danguir J (1995). Increased fat oxidation during Ramadan fasting in healthy women: an adaptative mechanism for body-weight maintenance. *The American Journal of Clinical Nutrition*.

[11] Hosseini SRA, Hejazi K (2013). The effects of Ramadan fasting and physical activity on blood hematological-biochemical parameters. *Iranian Journal of Basic Medical Sciences*.

[12] Furuncuoglu Y, Karaca E, Aras S, Yönem A (2007). Metabolic, biochemical and psychiatric alterations in healthy subjects during Ramadan. *Pakistan Journal of Nutrition*.

[13] Shariatpanahi VZ, Shariatpanahi VM, Shahbazi S, Hossaini A, Abadi A (2008). Effect of Ramadan fasting on some indices of insulin resistance and components of the metabolic syndrome in healthy male adults. *British Journal of Nutrition*.

[14] Lamri-Senhadji MY, El Kebir B, Belleville J, Bouchenak M (2009). Assessment of dietary consumption and time-course of changes in serum lipids and lipoproteins before, during and after Ramadan in young Algerian adults. *Singapore Medical Journal*.

[15] Ünalacak M, Kara IH, Baltaci D, Erdem Ö, Bucaktepe PGE (2011). Effects of ramadan fasting on biochemical and hematological parameters and cytokines in healthy and obese individuals. *Metabolic Syndrome and Related Disorders*.

[16] National Institutes of Health Consensus Development Panel (1985). Health implications of obesity. National institutes of health consensus development conference. *Annals of Internal Medicine*.

[17] Hill AJ, Blundell JE (1986). Macronutrients and satiety: the effects of a high-protein or high-carbohydrate meal on subjective motivation to eat and food preferences. *Nutrition and Behavior*.

[18] Stunkard AJ, Messick S (1985). The three-factor eating questionnaire to measure dietary restraint, disinhibition and hunger. *Journal of Psychosomatic Research*.

[19] Doucet E, St-Pierre S, Alméras N, Tremblay A (2003). Relation between appetite ratings before and after a standard meal and estimates of daily energy intake in obese and reduced obese individuals. *Appetite*.

[20] Kissileff HR (1984). Satiating efficiency and a strategy for conducting food loading experiments. *Neuroscience and Biobehavioral Reviews*.

[21] Green SM, Delargy HJ, Joanes D, Blundell JE (1997). A satiety quotient: a formulation to assess the satiating effect of food. *Appetite*.

[22] Drapeau V, Blundell J, Gallant AR (2013). Behavioural and metabolic characterisation of the low satiety phenotype. *Appetite*.

[23] Drapeau V, King N, Hetherington M, Doucet E, Blundell J, Tremblay A (2007). Appetite sensations and satiety quotient: predictors of energy intake and weight loss. *Appetite*.

[24] Friedewald WT, Levy RI, Fredrickson DS (1972). Estimation of the concentration of low-density lipoprotein cholesterol in plasma, without use of the preparative ultracentrifuge. *Clinical Chemistry*.

[25] Maughan RJ, Bartagi Z, Dvorak J, Zerguini Y (2008). Dietary intake and body composition of football players during the holy month of Ramadan. *Journal of Sports Sciences*.

[26] Leibel RL, Rosenbaum M, Hirsch J (1995). Changes in energy expenditure resulting from altered body weight. *The New England Journal of Medicine*.

[27] Finlayson G, Cecil J, Higgs S, Hill A, Hetherington M (2012). Susceptibility to weight gain. Eating behaviour traits and physical activity as predictors of weight gain during the first year of university. *Appetite*.

